# Factors associated with cognitive impairment in elderly versus nonelderly patients with metabolic syndrome: the different roles of FGF21

**DOI:** 10.1038/s41598-018-23550-9

**Published:** 2018-03-26

**Authors:** Arintaya Phrommintikul, Piangkwan Sa-nguanmoo, Jirapas Sripetchwandee, Prin Vathesatogkit, Nipon Chattipakorn, Siriporn C. Chattipakorn

**Affiliations:** 1 Department of Internal Medicine, Faculty of Medicine, Chiang Mai University, Chiang Mai, Thailand; 2 Neurophysiology Unit, Cardiac Electrophysiology Research and Training Center, Faculty of Medicine, Chiang Mai University, Chiang Mai, Thailand; 3 Department of Physiology, Faculty of Medicine, Chiang Mai University, Chiang Mai, Thailand; 4 Department of Medicine, Faculty of Medicine, Ramathibodi hospital, Mahidol University, Bangkok, Thailand; 5 Department of Oral Biology and Diagnostic Sciences, Faculty of Dentistry, Chiang Mai University, Chiang Mai, Thailand

## Abstract

Increased fibroblast growth factor 21 (FGF21) levels have been found in patients with metabolic syndrome (MetS). MetS is also associated with cognitive decline. However, the correlation between FGF21 and cognitive decline in elderly and nonelderly MetS patients has not been investigated. 116 non-elderly patients (age <65 years old) and 96 elderly patients (≥65 years old) with MetS were enrolled. Blood samples for FGF21 were collected from all participants after 12-hour fasting. Cognitive function was assessed using the Montreal cognitive assessment (MoCA) test. The MoCA score was negatively associated with age and was different among different levels of education in these MetS patients. In the non-elderly group, body mass index (BMI) showed positively correlated with MoCA score while, FGF21 level and HbA1C were negatively associated with the MoCA score in non-elderly MetS patients. BMI was the only factor which showed a negative correlation with the MoCA score in elderly MetS patients. This study demonstrated that FGF21 level was independently associated with cognitive impairment in non-elderly patients but not in elderly patients. The possible role of FGF21 level in cognitive impairment in non-elderly should be confirmed in a prospective study.

## Introduction

Metabolic syndrome (MetS) is a condition which involves an aggregation of atherosclerotic risk factors, leading to cardiovascular diseases such as ischemic heart disease and stroke. There is mounting evidence suggesting that the prevalence of MetS has significantly increased among the elderly and also that the aging process can lead to metabolic abnormalities^1–3^. Elderly patients with metabolic syndrome have both non-modifiable atherosclerotic risk factors of aging as well as modifiable metabolic risk factors^4,5^. Atherosclerotic risk factors, such as diabetes or hypertension, have been shown to be associated with cognitive dysfunction^6,7^. Metabolic syndrome in itself, as an aggregation of cardiovascular risk factors, is associated with cognitive impairment, Alzheimer’s disease (AD) and dementia^8–10^.

Cognitive impairment contributes to impairment in the quality of life. In addition, the resulting cognitive dysfunction can result in a lack of adherence to treatment, leading to high morbidity and mortality^11,12^. Due to an increasing prevalence of MetS, understanding the pathophysiology associated with cognitive impairment is essential in attempts to decrease the incidence of cognitive impairment.

It has been shown that impaired fasting glucose (IFG), an important MetS component, is strongly correlated with cognitive dysfunction in adults^13^. Previous studies have also demonstrated that MetS is associated with abnormality of memory, visuospatial abilities, executive functioning, processing speed, and overall intellectual functioning^10,14–16^.

The Montreal Cognitive Assessment (MoCA), a simple, stand-alone and rapid cognitive screening test, has been shown to have high sensitivity and specificity for the detection of mild cognitive impairment^17^. It enables the assessment of different cognitive domains: attention and concentration, executive functions, memory, language, visuoconstructional skills, conceptual thinking, calculations, and orientation^18^. The high sensitivity and specificity of this test made it a natural choice for use in this study. Although several factors could affect cognitive function in adults, aging can contribute to a dramatic decline in cognitive function. Currently, the correlation between metabolic parameters and the MoCA score in elderly and non-elderly MetS patients is not clear and has not been investigated.

Fibroblast growth factor 21 (FGF21) is an endocrine hormone that plays an important role in metabolic regulation. Interestingly, FGF21 levels have been found to be increased in conditions such as obesity, MetS and diabetes in both animal studies and clinical reports^19–22^. Several clinical studies have investigated the level of FGF21 in cases of human metabolic diseases such as MetS, impaired glucose tolerance and type 2 diabetes mellitus (T2DM), and have shown that increased FGF21 levels have a positive correlation with BMI, waist circumference, body fat mass, plasma insulin levels, triglycerides levels, and HOMA-IR, and a negative correlation with HDL and adiponectin levels in MetS patients^23–26^. A recent study found that FGF21 levels could be a biomarker for MetS^27^. However, the correlation between FGF21 levels and MoCA scores in elderly and non-elderly MetS patients have not yet been investigated. Thus, this study aimed to test the hypothesis that FGF21 levels are associated with cognitive performance in non-elderly and elderly MetS patients.

## Results

Of the 212 MetS patients enrolled onto the study, there were 116 non-elderly patients and 96 elderly patients. The non-elderly MetS patients had higher BMI, lower systolic blood pressure, higher HbA1C levels, and a higher prevalence of being a current smoker (Table 1). The mean age was 58.06 ± 4.56 vs. 71.78 ± 5.51 years in the non-elderly and elderly groups, respectively (p < 0.001). The mean MoCA score was 20.05 ± 4.66 vs. 18.43 ± 4.21 in the non-elderly and elderly groups, respectively (p < 0.05). The MoCA score was negatively associated with age (r = −0.17, p = 0.002). For the multilinear regression analysis with entry method of all 212 participants, we found that the factors negatively associated with MoCA score were age (B = −0.167, P = 0.011), HbA1C (B = −0.188, P = 0.011) and FGF21 level (B = −0.144, P = 0.042). In addition, all 212 participants in the present study had similar duration of fasting periods (8 hours). This same period of fasting state in all participants should exclude the variation of FGF21 levels from the fasting state in the present study. In addition, a previous study demonstrated that short fasting (overnight fasting) did not increase serum FGF21 levels^28^.

**Table 1:** Demographic data of all patients the comparison between non-elderly and elderly metabolic syndrome patients.

**Parameters**	**Non-elderly (N = 116)**	**Elderly (N = 96)**	**p value**
**Age (years)**	**58.06 ± 4.56**	**71.78 ± 5.51**	**<0.001**
Male (%)	52 (44.8%)	44 (45.8%)	0.884
**BMI (kg/m** ^**2**^ **)**	**28.72 ± 5.84**	**26.12 ± 4.85**	**0.001**
Waist circumference (cm)	96.50 ± 12.84	93.93 ± 12.80	0.148
Diabetes mellitus	99 (85.3%)	83 (86.5%)	0.817
Hypertension	106 (91.6%)	87 (90.6%)	0.848
Dyslipidemia	103 (88.8%)	86 (89.6%)	0.854
Current smoking	9 (7.8%)	0	0.003
Coronary artery disease	36 (31%)	29 (30.2%)	0.897
Cerebrovascular disease	42 (36.2%)	34 (35.4%)	0.905
Peripheral arterial disease	0	2 (2.1%)	0.118
**Systolic blood pressure (mmHg)**	**137.21 ± 18.14**	**143.33 ± 19.42**	**0.019**
**Diastolic blood pressure (mmHg)**	**75.79 ± 9.98**	**73.39 ± 9.38**	**0.074**
**HbA1C (%)**	**7.64 ± 1.78**	**7.02 ± 1.23**	**0.007**
**LDL-C (mg/dl)**	**100.60 ± 38.30**	**90.14 ± 33.85**	**0.052**
HDL-C (mg/dl)	49.94 ± 14.14	49.75 ± 16.71	0.723
Triglyceride (mg/dl)	121.59 ± 53.72	139.24 ± 80.74	0.106
HOMA index	2.65 ± 2.99	3.39 ± 7.07	0.749
FGF21 level (pg/ml)	399.08 ± 460.52	484.59 ± 764.88	0.328
**MOCA score**	**20.05 ± 4.66**	**18.43 ± 4.21**	**0.010**

BMI: body mass index; FGF21: fibroblast growth factor 21; HbA1C: Glycated hemoglobin A1C; HDL-C: high density lipoprotein cholesterol; HOMA index: Homeostasis Model Assessment index; LDL-C: low density lipoprotein cholesterol.

### Factors associated with MoCA score in non-elderly MetS patients

**Figure 1:**
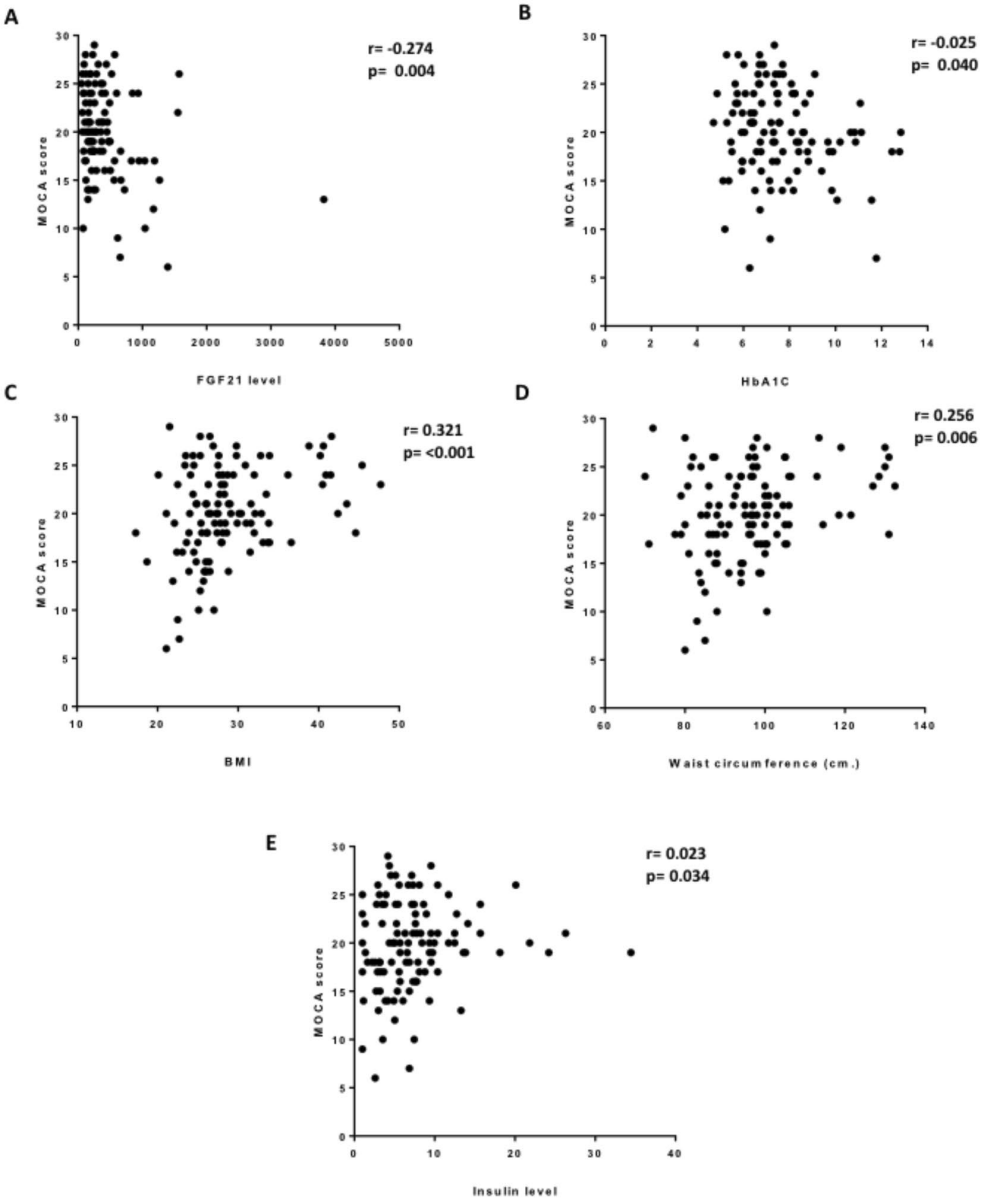
Factors associated with MoCA score in non-elderly metabolic syndrome patients. (**A**) FGF21, (**B**) HbA1c, (**C**) BMI, (**D**) waist circumference and (**E**) insulin.

Of the 116 non-elderly MetS patients: 3 (2.6%); 71 (61.2%); 42 (36.2%) received no education; primary or secondary school education, college or higher degree of education, respectively. There was a significant difference in MoCA score between the different levels of education (11.67 ± 5.13 vs. 18.76 ± 4.32 vs. 22.81 ± 3.46, p < 0.001) for none vs. primary or secondary school vs. college or higher degree, respectively. 64 (55.2%) of the cohort were male and 52 (44.8%) female. There was no difference in MoCA score between male and female (24.4 ± 4.24 vs. 19.76 ± 4.99, p = 0.277). Factors associated with MoCA score are presented in Table 2. FGF21 level and HbA1C were negatively associated with MoCA score (r = −0.274, p = 0.004 and r = −0.025, p = 0.40) respectively (Fig. 1A,B). BMI, waist circumference and insulin showed a positive correlation with MoCA score (r = 0.321, p < 0.001, r = 0.256, p = 0.006 and r = 0.023, p = 0.034 respectively) (Fig. 1C–E). After adjustment with education level in a multivariate analysis, FGF21, BMI and HbA1C level were independently correlated with the MoCA score (Table 3). The multivariate regression analysis included only either BMI or waist circumference demonstrated that FGF21 was the independent factor negatively associated with the MOCA score (B = −0.295, P = 0.002 or B = −0.287, P = 0.003, included only BMI or included only waist circumference, respectively). Due to the possibility of a correlation between factors associated with the MoCA score, the multicollinearity index was also analyzed and the Variance Inflation Factor (VIF) showed non-significant correlation among factors. There were 5 patients in this population who had had a stroke prior to the study. There was no difference in MoCA score between patients with and without a prior history of stroke (21.20 ± 5.07 vs. 20.00 ± 4.66, p = 0.576). In the analysis, excluding patients with a history of stroke, there was the significant negative association between MoCA score and FGF21 (r = −0.282, p = 0.003), and between MoCA score and HbA1C (r = −0.220, p = 0.02), and a significant positive association between MoCA score and BMI (r = 0.354, p = 0.05).

**Table 2:** Factors associated with MoCA score in non-elderly metabolic syndrome patients.

**Parameters**	**r**	**p (univariate)**
**FGF21**	**−0.274**	**0.004**
**BMI**	**0.321**	**<0.001**
**Waist circumference**	**0.256**	**0.006**
SBP	−0.087	0.353
Pulse pressure	0.023	0.808
**HbA1C**	−**0.205**	**0.040**
HOMA index	0.074	0.447
**Insulin**	**0.023**	**0.034**
Triglyceride	−0.017	0.870
HDL-C	−0.085	0.391
LDL-C Creatinine	−0.057 −0.146	0.567 0.129

BMI: body mass index; FGF21: fibroblast growth factor 21; HbA_1_C: Glycated hemoglobin A1C; HDL-C: high density lipoprotein cholesterol; HOMA index: Homeostasis Model Assessment index; LDL-C: low density lipoprotein cholesterol; MoCA: Montreal cognitive assessment; SBP: systolic blood pressure.

**Table 3:** FGF21, BMI and HbA1C level were independently correlated with the MoCA score after adjustment with education levels in a multivariate analysis.

Parameters	B	p	VIF
**BMI**	**0.356**	**0.036**	**3.431**
**HbA1C**	**−0.226**	**0.015**	**1.010**
Insulin Waist circumference	**−**0.156 **−**0.144	0.093 0.393	1.039 3.450
**FGF21**	**−0.277**	**0.003**	**1.005**

BMI: body mass index; FGF2: fibroblast growth factor 21; HbA_1_C: Glycated hemoglobin A1C; HDL-C: high density lipoprotein cholesterol; HOMA: index Homeostasis Model Assessment index; LDL-C: low density lipoprotein cholesterol; SBP: systolic blood pressure.

Medication being taken by the non-elderly MetS patients included: beta blockers; angiotensin converting enzyme inhibitors (ACEI); angiotensin receptor blockers (ARB); calcium channel blockers; diuretics; thiazolidinedione (TZD); fibrates and statins in the following proportions: 49.1%; 31.9%; 37.9%; 47.4%; 30.2%; 9.5%; 16.4% and 84.5%, respectively. There were no significant differences in the MoCA score between patients receiving and not receiving the prescribed medication (p > 0.05 in all medication).

### Factors associated with MoCA score in elderly MetS patients

Out of the 96 elderly MetS patients, 1 (1.0%), 68 (70.8%), 27 (28.1%) received no education, primary or secondary school education, college or higher degree of education, respectively. There was a significant difference in the MoCA score between the different levels of education (13.0 vs. 17.49 ± 4.22 vs. 20.89 ± 3.04, p < 0.001) for none vs. primary or secondary school vs. college or higher degree, respectively). There was no difference in the MoCA score between male and female (18.48 ± 4.60; 18.39 ± 3.88, p = 0.90). There were 8 patients with a prior history of stroke in this population. Patients with a prior stroke history had a lower MoCA score than patients with no such history (15.13 ± 5.19 vs. 18.74 ± 4.00, p = 0.019). Factors associated with MoCA score in elderly MetS patients are presented in Table 4. BMI showed a positive correlation with the MoCA score while serum creatinine level was negatively associated with MoCA score in elderly MetS patients. FGF21 level showed no correlation with MoCA score in elderly MetS patients. After multivariate analysis and adjustment for prior stroke, BMI was the only parameter positively associated with MoCA score (B = 0.206, p = 0.047).

**Table 4:** Factors associated with MoCA score in elderly metabolic syndrome patients.

Parameters	r	p (univariate)
FGF21	0.125	0.244
**BMI**	**0.218**	**0.036**
Waist circumference	0.196	0.060
SBP	−0.018	0.082
Pulse pressure	0.130	0.215
HbA1C	−0.098	0.369
HOMA index Insulin	−0.021 −0.034	0.848 0.75
Triglyceride level	−0.096	0.396
HDL-C	−0.011	0.916
LDL-C **Creatinine**	−0.195 −**0.228**	0.068 **0.028**

FGF21: fibroblast growth factor 21; BMI: body mass index; SBP: systolic blood pressure; HbA1C: Glycated hemoglobin A1C; HOMA: index Homeostasis Model Assessment index; HDL-C: high density lipoprotein cholesterol; LDL-C: low density lipoprotein cholesterol.

Among elderly MetS patients the following medication beta blockers, ACEI, ARB, calcium channel blockers, diuretics, TZD, fibrates and statins were prescribed in 47.9%, 25.0%, 52.1%, 51.2%, 44.8%, 10.4%, 8.3% and 84.4% of cases respectively. There was no significant difference of the MoCA score between patients with and without the medication (p > 0.05 in all medications).

With sample size of 116 non-elderly patients and the correlation coefficient of −0.274, a power to detect correlation between FGF21 and MOCA score was 84%. However, because the weak correlation between FGF21 and MOCA score (R = 0.125) in elderly patients, the power to detect significant correlation was 22.6%.

## Discussion

The major findings of this study show that the metabolic factors associated with MoCA score were different between elderly and non-elderly MetS patients. FGF21 level showed an independent negative association with the MoCA score only in non-elderly MetS patients. This suggests that there is a greater positive correlation between FGF21 and the severity of cognitive impairment in those patients than any other metabolic parameters on the severity of cognitive impairment in those patients. In addition, BMI, waist circumference and HbA1C level showed a significant positive correlation with MoCA score in non-elderly MetS patients. In elderly MetS patients, the only factor showing a positive correlation with the MoCA score was BMI.

Our study demonstrated an association between metabolic parameters and MetS in non-elderly patients independent of education level. These included FGF21, HbA1C level, BMI and MoCA score. We did not include gender in the multivariable analysis, because there was no difference in the MoCA score between male and female groups. In contrast, FGF21 was not associated with MoCA score in elderly MetS patients. Our study showed that elderly MetS patients had a lower MoCA score than the non-elderly MetS patients. These findings were consistent with a previous study which demonstrated that age significantly contributed to the predicted MoCA score or cognition^29^. Among these elderly MetS patients, other factors except education and BMI had no significant association with MoCA score. Although several studies showed that MetS or its components had no association with cognitive function in the elderly^30–32^, a recent study demonstrated that increased MetS components was positively associated with cognitive decline in individuals aged ≥60 years old^33^. That study by Tsai and colleagues showed that the most strongly associated factors with cognitive decline in MetS patients, measured using the digit symbol substitution test (DSST), were high plasma glucose and elevated blood pressure^33^. Interestingly, our findings showed that only BMI was associated with MoCA score in elderly MetS patients (aged ≥65 years old). The possible explanation for different findings between Tsai’s study and ours may be due to the differences in ages of elderly and the cognitive evaluation.

Unlike findings in elderly MetS patients, it has been shown that several metabolic parameters had a significant association with MoCA score in non-elderly or young elderly MetS patients^32,34^. Consistent with those reports, our results showed a significant association between metabolic parameters and MoCA score. In addition, our study demonstrated for the first time that FGF21 level was negatively associated with MoCA score in only non-elderly MetS patients. It is known that MetS can cause an increase in FGF21 level^19^. The changes in FGF21 level have also been shown to be dependent on age in healthy adults^35^. Hanks and colleagues showed that FGF21 levels increase with age independently of body composition^35^. However, our study showed that FGF21 level between elderly and non-elderly MetS patients was not significantly different. FGF21 levels tended to be higher in elderly MetS patients when compared to the non-elderly group. The possible explanation of the different findings between our study and Hanks’s study could be that all subjects in our study were MetS patients in which FGF21 levels were already increased. Since the level of FGF21 is already high in MetS patients and age can also lead to increased FGF 21 level, then it could be difficult to show the association between FGF21 level and cognition in elderly MetS patients. Therefore, the change in FGF21 level could be associated with MoCA score in non-elderly MetS patients, rather than the elderly group. The association of cognitive function and metabolic profiles was not observed across the entire age range by using regression. This could be due to: (1) factors associated with cognitive function may be different between the elderly and non-elderly groups; and (2) the degenerative process in the elderly group might play a stronger role than metabolic disturbances in the modulation of cognitive function^30^.

Previous studies demonstrated that long-term use of peroxisome proliferator-activated receptor (PPAR)-α agonist, such as fibrate or a peroxisome proliferator-activated receptor (PPAR) γ agonist, such as thiazolidinedione (TZD), might have an impact on circulating FGF21 levels^36,37^. In our study, we found that FGF21 level was significantly higher in patients receiving fibrate than patients without fibrate (726.1 ± 958.5 vs. 407.6 ± 564.8 pg/ml, p = 0.009). However, FGF21 level was not significantly difference between patients with TZD and those without TZD (302.0 ± 217.3 vs. 455.9 ± 649.9 pg/ml; p = 0.346). In addition, the MoCA score was not different between patients with and without fibrate or TZD. We also carried out the data analysis between the MoCA score and the FGF21 level without excluding patients receiving PPARα- and PPARγ-agonists. We found that the MoCA score still showed a statistically significant negative correlation with the FGF21 level (r = −0.285, p = 0.007) in non-elderly MetS patients.

Similarly to the findings of our previous study, which demonstrated that FGF21 level crucially depends on renal function^38^, our study showed an association between serum creatinine and FGF21 (r = 0.497, p < 0.001). However, there was no correlation between serum creatinine level and MoCA score and the associations between variables associated with MoCA score were preserved after adjustment with the serum creatinine level.

Although it has been shown previously that adiponectin can be a crucial mediator in the metabolic effects of FGF21 levels in mice^39^, we did not determine the adiponectin level in this study. Future studies are needed to determine the association between the levels of adiponectin and FGF21 in MetS patients. The association between BMI and cognitive function are still controversial. Some studies have shown a negative association between BMI and cognitive function in young adults^40^, while some studies showed a positive association between BMI and cognitive function in the middle aged or elderly populations^41,42^. Our study demonstrated a positive correlation between BMI and MoCA in both this elderly and non-elderly population. The discrepancies in the association between BMI and cognitive function may be contributed to by the interaction between medical conditions such as cardiovascular disease and also the global health status on BMI^42^. Patients with multiple atherosclerotic risk factors, as well as established cardiovascular disease, in the population of the present study suggest that a lower BMI may reflect lower health status and a lower nutritional status, finally leading to a lower MoCA score or cognitive decline.

The effects of medication commonly prescribed in MetS patients on cognitive function were also explored in our study and our findings suggest that the medication taken by the MetS patients in this cohort had no effect on cognitive function.

The limitations in the present study were: (1) the study population was relatively small; (2) the metabolic parameters associated with cognitive function may have interactions with other parameters. To attempt to address the second limitation the multicollinearity index was also calculated, and the VIF showed insignificant interaction; and (3) the multivariate regression analysis of the present study contains confounding variables, such as BMI and waist circumference. Unfortunately, the hip circumference in those patients was not measured in the present study, and therefore the waist/hip ratio (WHR) could not be determined. Future study is needed to investigate the association between the WHR and the MoCA score in MetS patients.

In summary, this study indicates that FGF21 level, HbA1c and BMI are factors which are facors associated with the MoCA score in non-elderly MetS patients. The only factor associated with MoCA score in elderly MetS patients was the BMI. The findings of this study suggest that increased FGF21 in non-elderly MetS patients is associated with cognitive decline. The possible role of FGF21 as a risk factor of cognitive impairment should be further explored in a prospective study.

## Subjects and Methods

The study protocol was reviewed and approved by the institutional Ethics Committee of the Faculty of Medicine, Chiang Mai University, Chiang Mai, Thailand. All patients gave written informed consent for participation in this research. All methods were performed in accordance with the relevant guidelines and regulations. The present study is a sub-study of MetS patients in The Cohort Of patients at a high Risk for Cardiovascular Events (CORE) - Thailand registry. The CORE-Thailand registry is an ongoing prospective study involving a cohort of Thai patients with high atherosclerotic risks. Patients aged 45 years or older with established coronary artery disease (CAD), cerebrovascular disease (CVD), or peripheral arterial disease (PAD), or with at least 3 atherosclerosis risk factors (multiple risk factors, MRF) were enrolled from the outpatient clinic at Maharaj Nakorn Chiang Mai Hospital in the period April 2011 to March 2014. Patients with the following conditions were excluded from the cohort: (1) patients who had had an acute atherosclerotic event within 3 months; (2) patients who had a large aortic aneurysm indicated for surgery; (3) patients who participated in a blind clinical trial; (4) patients who had a limited life expectancy from a non-cardiovascular condition such as cancer or a documented human immunodeficiency virus (HIV) infection, or (5) those who might have difficulty returning for a follow-up visit. Patients who met 3 out of 5 criteria for MetS were invited to participate in this study. The criteria used to assess for MetS included: (1) elevated waist circumference (≥90 cm in men and ≥80 cm in women); (2) blood triglyceride level ≥150 mg/dl or treated; (3) high density lipoprotein cholesterol (HDL-C) <40 mg/dl in men and <50 mg/dl in women; (4) blood pressure ≥130/85 mmHg or treated; (5) fasting glucose ≥100 mg/dl or treated^43^. Dataset of MetS patients were split into two groups: nonelderly (<65 years old) and elderly (≥65 years old) groups. This cut-off age was chosen due to the fact that it has been used previously as regards cardiovascular guidelines^44^. In addition, this specific age cut-off (65 years old) was also used in our previous study and the use would give consistency between the reports^45^.

### Data collection

History taking, physical examination, blood sampling and cognitive function assessment were performed on the same day. Cognitive function was assessed by the same investigator using The Montreal Cognitive Assessment (MoCA).

### Montreal Cognitive Assessment (MoCA)

Mild cognitive impairment was assessed using the Montreal Cognitive Assessment (MoCA). It enables the assessment of different cognitive domains including attention and concentration, executive function, memory, language, visuoconstructional skills, conceptual thinking, calculation, and orientation. The total score is 30 points; a score of 26 or above is considered normal^18^. Since the MoCA score has a higher diagnostic performance than the Mini–Mental State Examination (MMSE score) for mild cognitive impairment this study used the MoCA score for the analysis of cognitive function^46^.

### Chemical analysis

**Figure 2:**
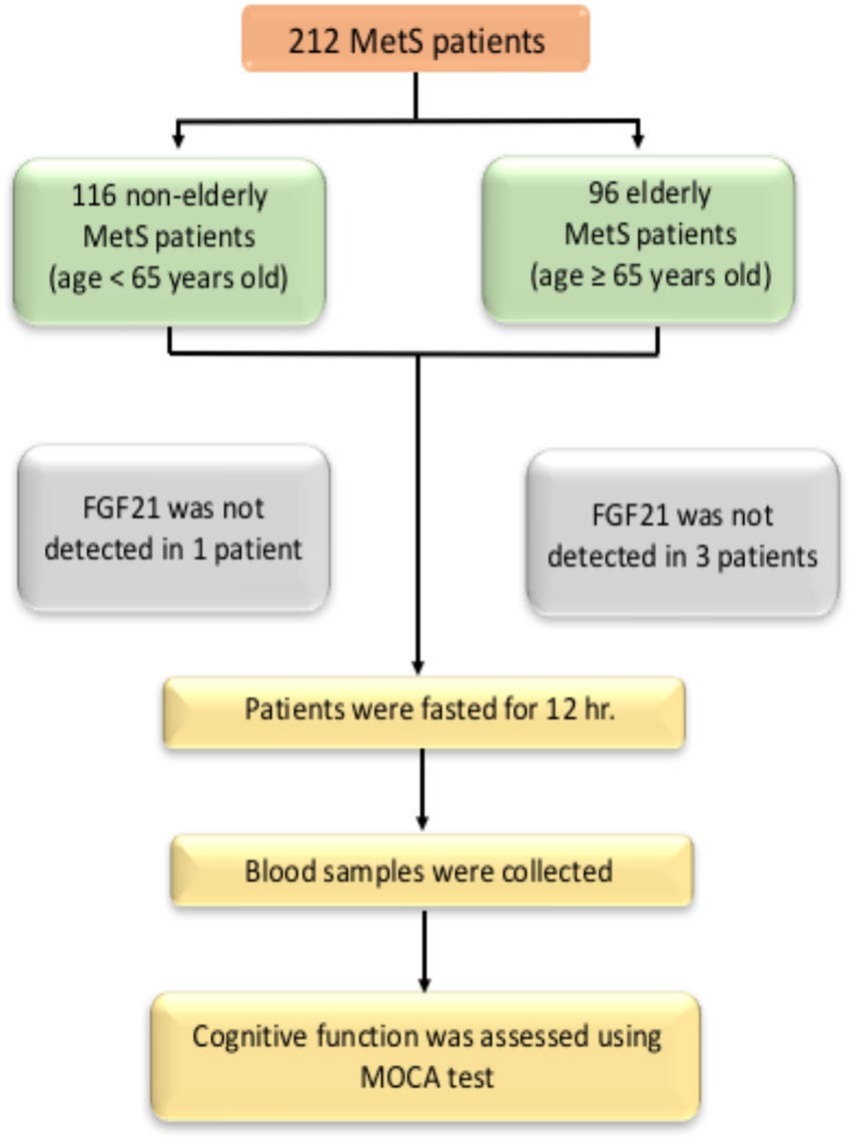
The experimental protocol of the present study.

Fasting blood samples were obtained from all participants enabling the calculation of the fasting plasma glucose, HDL-C, LDL-C, triglyceride, insulin and FGF21 levels. Fasting plasma glucose and triglyceride levels were determined by colorimetric assay (ERBA diagnostic, Mannheim, Germany). Fasting plasma insulin levels were evaluated using a sandwich enzyme-linked immunosorbent assay (ELISA) kit (Millipore, MI, USA). Plasma FGF21 levels were determined using a human FGF21 enzyme-linked immunosorbent assay (ELISA) kit (R&D systems Inc., Minneapolis, MN, USA). The severity of peripheral insulin resistance was assessed using the homeostasis model assessment (HOMA) as described previously^47^. Fasting plasma glucose level was used to calculate the HOMA index. FGF21 levels were not detectable in 4 MetS patients, 1 non-elderly patient and 3 elderly patients. This data was described as missing data in the analysis as shown in Fig. 2.

### Statistical analysis

Continuous variables are expressed as mean ± SD. Categorical variables are expressed as frequencies and percentages and comparison between groups was made using the Pearson χ2 test. The metabolic profiles in the study consisted of the following normally distributed variables: age, body mass index, waist circumference, LDL-C, HDL-C, and the non-normally distributed variables: HOMA index, and FGF21, triglyceride, and insulin levels. The Student’s t-test and nonparametric test (Mann-Whitney U Test) were used in the analysis of the normally distributed variables and non-normally distributed variables, respectively. The correlation between the association of factors with the MoCA score was analyzed using the multicollinearity index. Due to the significant impact of aging on cognitive function, the patients were classified into non-elderly and elderly groups using the age of 65 or older as the classification for elderly. Statistical significance was considered as a 2-tailed probability of less than 0.05. All statistical calculations were assessed using commercially available software (SPSS version 22, SPSS Inc., Chicago, IL, USA).
